# Thoracoabdominal Asynchrony Contributes to Exercise Limitation in Mild Asthmatic Subjects

**DOI:** 10.3389/fphys.2018.00719

**Published:** 2018-06-13

**Authors:** Guilherme Fregonezi, Antonio Sarmento, Janaína Pinto, Antonella LoMauro, Vanessa Resqueti, Andrea Aliverti

**Affiliations:** ^1^PneumoCardioVascular Lab, Hospital Universitário Onofre Lopes, Empresa Brasileira de Serviços Hospitalares, Departamento de Fisioterapia, Universidade Federal do Rio Grande do Norte, Natal, Brazil; ^2^Dipartimento di Elettronica, Informazione e Bioingegneria, Politecnico di Milano, Milan, Italy

**Keywords:** asthma, breathing pattern, dynamic hyperinflation, post-inspiratory action, exercise

## Abstract

This study aimed to better understand how subjects with stable asthma and without exercise-induced bronchoconstriction respond to mild exercise. Breathing pattern, chest wall compartmental and operational volumes, and thoracoabdominal asynchrony were assessed in 11 stable asthmatic subjects and 10 healthy subjects at rest and during exercise in a cycle-ergometer through optoelectronic plethysmography. Dyspnea and sensation of leg effort were assessed through Borg scale. During exercise, with similar minute ventilation, a significant lower chest wall tidal volume (*p* = 0.003) as well as a higher respiratory rate (*p* < 0.05) and rapid shallow breathing (*p* < 0.05) were observed in asthmatic when compared to healthy subjects. Asthmatic subjects exhibited a significantly lower inspiratory (*p* < 0.05) and expiratory times (*p* < 0.05). Intergroup analysis found a significant higher end-expiratory chest wall volume in asthmatic subjects, mainly due to a significant increase in volume of the pulmonary ribcage (RCp; 170 ml, *p* = 0.002), indicating dynamic hyperinflation (DH). Dyspnea and sensation of leg effort were both significantly greater (*p* < 0.0001) in asthmatic when compared to healthy subjects. In addition to a higher thoracoabdominal asynchrony found between RCp and abdominal (AB) (*p* < 0.005) compartments in asthmatic subjects, post-inspiratory action of the inspiratory ribcage and diaphragm muscles were observed through the higher expiratory paradox time of both RCp (*p* < 0.0001) and AB (*p* = 0.0002), respectively. Our data suggest that a different breathing pattern is adopted by asthmatic subjects without exercise-induced bronchoconstriction during mild exercise and that this feature, associated with DH and thoracoabdominal asynchrony, contributes significantly to exercise limitation.

## Introduction

The maintenance of steady lung volumes during exercise in healthy subjects requires a fine coordination action between inspiratory and expiratory muscles. This coordination prevents ribcage distortion and makes the diaphragm acting as the main flow generator and the expiratory muscles to decrease abdominal volume and elevate pleural and alveolar pressures in order to decrease end-expiratory volume (EEV) (Henke et al., 1988; Aliverti et al., 1997). In addition, intra- and extra-thoracic airway dilatation occurs in order to minimize the increase of inspiratory muscles load (England and Bartlett, 1982; Warren et al., 1984) followed by an increase in both tidal volume and respiratory rate (Aliverti, 2008).

In patients with different lung diseases, different breathing pattern adjustments are necessary to cope with the ventilatory demands during exercise (Wilkens et al., 2010). In asthmatic subjects, even under stable conditions, exercise responses may vary (Del Giacco et al., 2015) due to an altered breathing pattern (i.e. increased respiratory rate, EEV, and rapid shallow breathing), thoracoabdominal asynchrony (Tobin et al., 1983; Hillman et al., 1986; Lavietes et al., 1988; Upton et al., 2012) and fluctuation in airway caliber (Barnes et al., 1981; Crimi et al., 2002; Kosmas et al., 2004; Ferreira et al., 2017), which may contribute to dyspnea and exercise limitation (Aliverti, 2008). However, differently from COPD (Aliverti et al., 2009; Priori et al., 2013; O’Donnell et al., 2017), little is known about the thoracoabdominal asynchrony and ventilatory responses in asthmatic subjects during exercise.

To the best of our knowledge, the breath-by-breath changes in compartmental operational volumes during exercise had not been studied in mild asthmatic subjects without exercise-induced bronchoconstriction. The aim of the present study was to study how mild asthmatic subjects without exercise-induced bronchoconstriction respond to mild exercise and the possible mechanisms leading to exercise limitation. For this, we assessed total and compartmental chest wall (CW) and operational volumes, breathing pattern and thoracoabdominal asynchrony in stable asthmatic subjects through optoelectronic plethysmography during exercise on a cycle-ergometer and compared with healthy subjects.

We hypothesized that in stable asthmatic subjects exercise induces dynamic hyperinflation (DH) and an altered breathing pattern and these features are associated with an uncoordinated respiratory muscle action.

## Materials and Methods

### Subjects

This is a cross-sectional study approved by institutional Research Ethics Committee in Brazil (number 521/2011) and the Ferrara University Ethics Committee in Italy (Aliverti et al., 2011), and performed in accordance with the Declaration of Helsinki. All subjects signed an informed consent form.

Outpatients with asthma diagnosed according to the Global Initiative for Asthma (Global Initiative for Asthma [GINA], 2017), aged between 18 and 50 years, were recruited from the Hospital Universitário Onofre Lopes (HUOL/EBSERH) in Brazil. The severity of the disease was also defined according to GINA guidelines (Global Initiative for Asthma [GINA], 2017). Patients under medical treatment for more than 6 months and considered clinically stable (without asthmatic attack and hospitalization in the 6 months prior to the study or changes in medication for at least 30 days) without other chronic lung diseases which could cause or contribute to breathlessness were included in the study. Subjects with cardiovascular or musculoskeletal diseases that could limit their ability to perform the exercise protocol, as well as those who presented bronchoconstriction induced by exercise were excluded from the study. Control group was composed of healthy subjects recruited in a previous study (Aliverti et al., 2011) and were in large part, but not exactly, the same subjects whose data were published. Inclusion criteria for controls were no previous history of smoking, absence of cardiopulmonary disease, and forced vital capacity (FVC) and forced expiratory volume in the 1st s (FEV_1_) above 80% of predicted. None of the participants reported having a lifelong smoking status.

### Protocol

In asthmatic subjects, the assessments were carried out on two different days, with an interval of 7 days in between, by a trained physiotherapist. Subjects included adhered to the standard withdrawal of inhaled medications prior to each visit: short-acting bronchodilators (≥12 h), long-acting bronchodilators (≥24 h). Subjects were asked to avoid caffeine-containing beverages and alcohol and heavy meals at least 4–6 h prior to testing, as well as strenuous physical exertion for at least 12 h before each visit day (Laveneziana et al., 2013).

On the first day, anthropometric and pre- and post-bronchodilator spirometric assessments were performed. In the second day, the incremental cycle-ergometer test was carried out. Considering that the presence of bronchoconstriction induced by exercise could influence exercise capacity, spirometry was also performed prior and after 10 min of the exercise protocol (Parsons et al., 2013). In healthy subjects, the assessments were carried out on the same day, with suitable resting time between spirometric and incremental cycle-ergometer tests (Aliverti et al., 2011).

After positioning the markers for optoelectronic plethysmography (see below) in specific anatomical points of the thorax and abdomen, the subjects were asked to breathe comfortably while seated in a cycle-ergometer (Corival, Lode, Groningen, Netherlands for asthmatics and Ergoline S800, Bitz, Germany for controls) with arms supported away from the sides of the body to allow the visualization of the markers by the TV cameras. After 120 s of quiet spontaneous breathing (QB), subjects were asked to perform 60 s of unloaded cycling, and successively the workload was increased by 30 watts every 3 min until reach limited symptom maximal intensity. Heart rate and oxygen saturation were monitored during the entire protocol by a portable wrist pulse oximeter (Nonin^®^ 2500, PalmSat, Plymouth, United States) in Brazil and (Ohmeda, Helsinki, Finland) in Italy. At the end of each workload, the subjects were asked to rate their perceived level of exertion separately for respiratory and leg effort on a 10-point category rating Borg scale (Wilson and Jones, 1989). Maximum workload (W_max_) was defined as the highest work rate maintained for at least 30 s. In asthmatic subjects the test was interrupted when the feeling of dyspnea and/or lower limb fatigue reached an intensity of 7 points (according to Borg scale) or 80% of maximum heart rate (Tanaka et al., 2001), while in healthy the test was interrupted voluntarily by exhaustion (Aliverti et al., 2011).

### Measurements

#### Pulmonary Function

A DATOSPIR 120C spirometer (Sibelmed^®^, Barcelona, Spain) and a MasterScreen Body plethysmograph (MasterLab, Jaeger, Würzburg, Germany) were used in the asthmatic subjects in Brazil and healthy in Italy (Aliverti et al., 2011), respectively, to measure FEV_1_, FVC, and FEV_1_/FVC ratio. All measurements were obtained according to previous technical procedures and criteria of acceptability and reproducibility (American Thoracic Society/European Respiratory Society, 2002). The best curve obtained from three acceptable maneuvers was considered in the study. Values obtained were compared to absolute and predictive values for the Brazilian (Pereira et al., 2007) and Italian population (Quanjer et al., 1993).

#### Optoelectronic Plethysmography

Optoelectronic plethysmography (OEP-BTS^®^, Milan, Italy) was used to measure CW kinematics at rest and during exercise in both asthma and healthy. The system, composed of six infrared TV cameras, captured at 60 frames.sec^-1^ the movement of 89 reflecting markers placed over the thorax and abdomen (37 on the anterior ribcage, 42 on the posterior ribcage and 10 lateral markers, 5 on each side of the chest) (Aliverti and Pedotti, 2003) and volumes were obtained by applying Gauss Theorem (Cala et al., 1996).

### Data Analysis

#### Chest Wall Volumes and Kinematics

The volume of the CW was modeled as the sum of the volumes of the pulmonary-apposed ribcage (RCp), diaphragm-apposed ribcage (RCa), and abdomen (AB) (Ward et al., 1992). Tidal (V_T_), end-inspiratory volume (EIV), and EEV of both CW and different compartments were considered for data analysis. In addition, all ventilatory parameters including respiratory rate, minute ventilation, inspiratory and expiratory flow, inspiratory and expiratory time, duty cycle, and total respiratory cycle time were calculated form total CW volume tracings and analyzed. Rapid shallow breathing was obtained as the ratio between respiratory rate and tidal volume (Yang and Tobin, 1991). Volume variations of AB and RCp, divided by inspiratory time were used as indexes of shortening velocity index of diaphragm and inspiratory ribcage muscles, respectively, while volume variations of the AB divided by expiratory time as index of shortening velocity of abdominal expiratory muscles, as previously described (Aliverti et al., 2002).

#### Thoracoabdominal Asynchrony

Asynchrony was quantified by determining the phase angle (θ) between volume variations of RCp, RCa, and AB. θ was indicated by the degree of opening of the Lissajous figure, computed as θ = sin^-1^(m/s), when measuring the ratio of the distance (m) delimited by the intercepts of the *y*-axis and *x*-axis loop on a line parallel to the *x*-axis at 50% of the *y*-axis divided by *x*-axis (s) (Agostoni and Mognoni, 1966; Konno and Mead, 1967). V_RCp_ (*y*-axis) versus V_RCa_ (*x*-axis) and V_RCp_ (*y*-axis) *versus* V_AB_ (*x*-axis) plots were calculated at rest and during exercise. By convention a positive θ means that *y*-axis is leading on *x*-axis expansion; on the contrary, negative θ describes the reverse situation (Allen et al., 1990). Moreover, a θ of zero represents completely synchronous movement of the compartments and 180° total asynchrony (Aliverti et al., 2009).

In addition, inspiratory and expiratory paradox times of RCp (IP_RCp_ and EP_RCp_), RCa (IP_RCa_ and EP_RCa_), and AB (IP_AB_ and EP_AB_), defined as the fraction of time in which the V_RCp_, V_RCa_, and V_AB_ decrease during inspiration and increase during expiration, respectively, were calculated (Aliverti et al., 2009).

### Statistical Analysis

All data, here presented as mean ± SD unless otherwise stated, were analyzed and compared between asthmatic and controls at four different levels, i.e., rest and 0, 30, and 60 watts exercise. Starting from CW volume tracings, all breathing pattern, CW volume and asynchrony parameters were calculated from the last 5–10 breaths at the end of the period of QB and of each exercise workload. EEV and EIV of the CW and its compartments were reported as variations from the baseline volumes at FRC before subjects started pedaling. Data analysis and statistics were performed by a blinded researcher.

Normality and distribution of data were verified using Shapiro–Wilk test. As group sample sizes were not equal, distribution of data was confirmed using the Welch and Brown–Forsythe test. Anthropometric and spirometric data between groups (intergroup analysis) were compared using unpaired *t*-test and Mann–Whitney test. Pre- and post-bronchodilator and exercise spirometric data were compared using paired *t*-test. Two-way repeated measures ANOVA was performed when variables were normally distributed and, in the event of a statistically significant difference, Bonferroni’s *post hoc* test was applied in order to identify the difference between levels (intragroup analysis). When normality test failed, intra- and intergroup data were compared applying Friedman’s and Mann–Whitney tests, respectively.

Separate backward stepwise linear regression analyses models were built to assess predictors of FEV_1_ and dyspnea and sensation of leg fatigue (assessed using the Borg scale). The first two backward stepwise linear regression analysis was undertaken between FEV_1_ (in liters and percentage of predicted values) as dependent variables and asynchrony variables as well as CW volume variables (independent variables). The same analysis was performed to predict dyspnea and sensation of leg fatigue (as dependent variables) with asynchrony and CW volumes (independent variables).Intergroup effect-sizes were calculated for all parametric and non-parametric data. For the first, Cohen’s *f* was calculated according to Cohen (1988) and interpreted as low (<0.10), moderate (between 0.25 and 0.40), and high (>0.40). For the latter, Cohen’s *d* was calculated according to Fritz et al. (2012) and expressed as low (<0.20), moderate (between 0.20 and 0.50), and high (>0.80).

Effect-sizes and the power of the study were calculated (Cohen, 1988) using G^∗^Power 3.1.9.2 software (Franz Faul – Universität Kiel, Germany). Descriptive and inferential analyses were conducted using GraphPad Prism^®^ software (GraphPad Software Inc., La Jolla, United States). *P*-values lower than 0.05 were considered statistically significant.

## Results

Anthropometric characteristics and spirometric values are reported in **Table 1**. All subjects were characterized by mild asthma according to the severity of the disease. Asthmatic subjects showed a significantly lower (*p <* 0.0001) FVC and FEV_1_ when compared to controls and a significant higher (*p* < 0.05) FVC and FEV_1_ after the use of the bronchodilator. After bronchodilator reversibility test, 63 % of the asthmatic patients showed bronchodilator reversibility, i.e., an increase in FEV_1_ >12% and >200 mL from baseline after bronchodilator (Pellegrino et al., 2005). The patients who were not fully reversible during bronchodilator reversibility test (27% of the asthmatic patients) had a history of childhood asthma. W_max_ was equal to 60 watt for 9 out of 11 asthmatic subjects, and it was 90 and 150 for the other 2. In controls, W_max_ was equal to 150 watt for 5 subjects, 210 watts for 3 subjects, and 240 watts for the remaining 2.

**Table 1:** Anthropometric and spirometric values of healthy and asthmatic subjects.

	Healthy			Asthma			
Subjects_(n)_	10			11			
Male/Female_(n)_	10/0			7/4			
Age_(years)_	40.3 ± 12.5			34.4 ± 11			
Weight_(kg)_	76.3 ± 10.4			74.5 ± 14			
Height_(m)_	1.76 ± 0.04			1.73 ± 0.11			
BMI_(kg/m2)_	24.6 ± 3.3			26 ± 3.5			

		**Bronchodilator**			**Exercise**		
		**Pre**	**Post**	**% Δ**	**Pre**	**Post**	**%Δ**
		
FVC_(L)_	5.12 ± 0.35	4.02 ± 0.82^†^	4.26 ± 0.81*	+5.9	4.06 ± 0.71	4.16 ± 0.71	+3.2
FVC_(%pred)_	109 ± 14	85 ± 13^†^	92 ± 14*	+7.2	87 ± 13	89 ± 12	+3.1
FEV_1(L)_	4 ± 0.35	2.22 ± 0.61^†††^	2.40 ± 0.71*	+8.1	2.20 ± 0.63	2.27 ± 0.62	+3.1
FEV_1(%pred)_	107 ± 12	66 ± 17^†††^	72 ± 17*	+8.1	66 ± 15	68 ± 14	+2.7
FEV_1_/FVC	0.78 ± 0.08	0.55 ± 0.11	0.56 ± 0.13	2	0.54 ± 0.13	0.55 ± 0.01	0
FEV_1_/FVC_(%pred)_	96 ± 9	69 ± 10	70 ± 9	0	67 ± 13	69 ± 10	0

*Data presented as mean value and SD. FVC, Forced Vital Capacity; FEV_*1*_, forced expiratory volume in the 1st s; FEV_*1*_/FVC, ratio of forced expiratory volume in the 1st s to forced vital capacity; n, number of subjects; m, meters; kg, kilograms; L, liters; %pred, percentage of predicted; %Δ, percentage of change between pre and post; ^†^*p* < 0.05 compared with healthy; ^†††^*p* < 0.0001 compared with healthy; ^∗^*p* < 0.05 when compared with pre bronchodilator*.

### Ventilatory Parameters, Dyspnea and Sensation of Leg Effort

**FIGURE 1:**
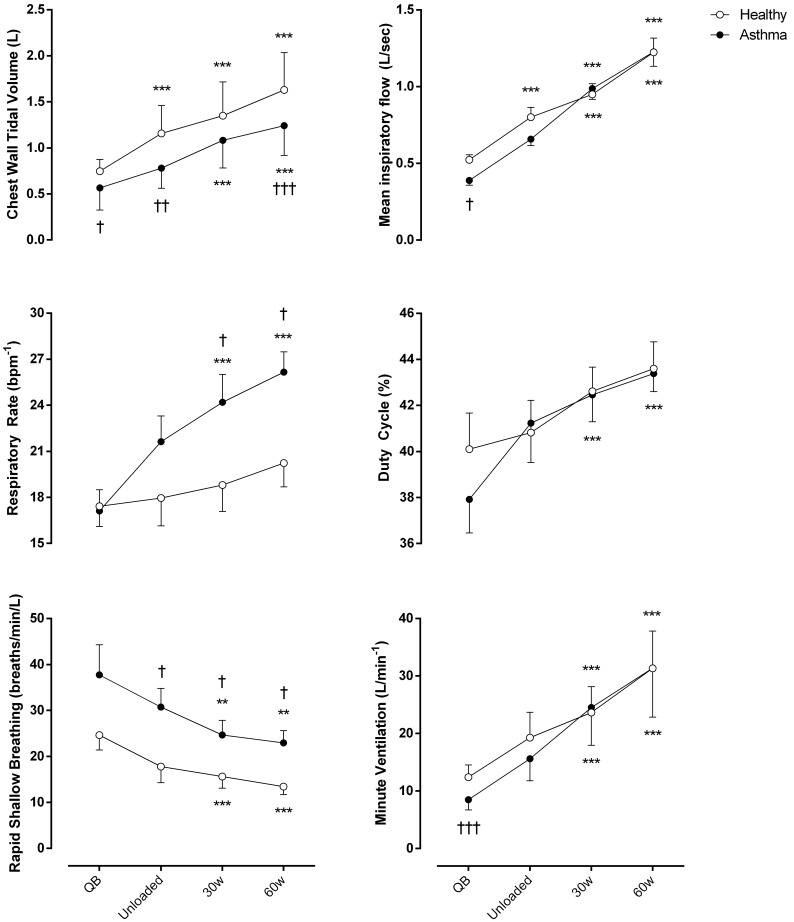
Ventilatory parameters of both asthmatic and healthy subjects during quiet breathing (QB), unloaded cycling, and 30 and 60 watts of workload. Data are shown as mean value and SE. L, Liters; Bpm^-1^, breaths per minute; Min, minutes; Sec, seconds; ^∗∗^*P* < 0.005 and ^∗∗∗^*P* < 0.001 compared to QB moment; ^†^*P* < 0.05, ^††^*P* < 0.005 and ^†††^*P* < 0.001 compared to healthy subjects.

As shown in **Figure 1**, compared to controls, minute ventilation was significantly lower in asthmatic at rest (*p* < 0.001, Cohen’s *f* = 1.05), due to a reduced tidal volume with similar respiratory rate. During exercise, at all the considered levels, minute ventilation became similar between asthmatics and controls with, however, a substantially different breathing pattern characterized by higher respiratory rate (Cohen’s *f* = 0.67) and lower V_T_ (Cohen’s *f* = 1.19), resulting in a higher rapid and shallow breathing pattern in asthmatics (Cohen’s *f* = 0.70).

As shown in **Table 2**, at resting conditions, expiratory flow (Cohen’s *f* = 0.93) and shortening velocity of diaphragm (Cohen’s *d* = 0.46) and expiratory muscles (Cohen’s *d* = 0.57) were significantly lower in asthmatics compared to controls. During exercise inspiratory time (Cohen’s *f* = 0.51), expiratory time (Cohen’s *f* = 0.53), and total respiratory cycle (Cohen’s *f* = 0.57) time were significantly lower in asthmatics at 30 and 60 watt compared to these variables in health subjects. On the other hand, baseline HR was higher in asthmatic when compared with healthy (Cohen’s *f* = 0.48) and increased in a similar way during the protocol in both groups.

**Table 2:** Ventilatory patterns, oxygen saturation and heart rate during quiet breathing and exercise.

	Healthy				Asthma			
	QB	Unload	30 watts	60 watts	QB	Unload	30 watts	60 watts
Ti _(sec)_ ‡	1.52 [1.16–1.87]	1.44 [1.07–1.59]	1.38 [0.95–1.22]	1.36 [0.90–1.12]	1.26 [1.17–1.66]	1.10 [1.09–1.74]	1.06 [1.12–1.73]^∗∗∗,†^	1.06 [1.11–1.68]^∗∗∗,†^
Te _(sec)_ ‡	2.25 [1.82–2.82]	2.28 [1.70–2.85]	1.99 [1.55–2.63]	1.86 [1.47–2.28]	2.56 [1.96–3.31]	1.89 [1.34–2.55]	1.65 [1.21–1.81]^∗∗∗,†^	1.45 [1.14–1.56]^∗∗∗,†^
Ttot _(sec)_ ‡	3.55 [3.26–4.51]	3.65 [2.77–4.52]	3.44 [2.77–4.27]	3.22 [2.51–3.81]	3.84 [3.22–4.75]	2.94 [2.39–4.15]	2.71 [2.27–3.34]^∗∗∗,†^	2.48 [2.05–2.61]^∗∗∗,†^
Exp. Flow _(L/sec)_	0.35 [0.28–0.41]	0.54 [0.45–0.66]	0.70 [0.59–0.80]^∗∗∗^	0.93 [0.74–1.13]^∗∗∗^	0.23 [0.19–0.29]^††^	0.46 [0.40–0.48]	0.73 [0.54–0.94]^∗∗∗^	0.88 [0.76–1.16]^∗∗∗^
V_RCp_/Ti _(L/sec)_ ‡	0.20 [0.13–0.21]	0.23 [0.17–0.21]	0.26 [0.24–0.30]^∗∗∗^	0.39 [0.30–0.43]^∗∗∗^	0.14 [0.08–0.17]	0.20 [0.16–0.22]^∗∗∗^	0.31 [0.30–0.36]^∗∗∗^	0.37 [0.30–0.49]^∗∗∗^
V_AB_/Ti _(L/sec)_	0.23 [0.19–0.31]	0.46 [0.27–0.51]^∗∗∗^	0.51 [0.36–0.57]^∗∗∗^	0.62 [0.42–0.71]^∗∗∗^	0.15 [0.14–0.24]^†^	0.33 [0.28–0.35]	0.45 [0.38–0.59]^∗∗∗^	0.54 [0.54–0.78]^∗∗∗^
V_AB_/Te _(L/sec)_	0.17 [0.08–0.11]	0.29 [0.17–0.31]^∗∗∗^	0.37 [0.23–0.45]^∗∗∗^	0.43 [0.37–0.57]^∗∗∗^	0.10 [0.13–0.20]^†^	0.22 [0.23–0.33]	0.36 [0.27–0.40]^∗∗∗^	0.42 [0.37–0.56]^∗∗∗^
%SpO_2_	98 [97.7–99]	98 [97–99]	98 [97–99]	98 [97–99]	98 [97–99]	98 [97–99]	98 [97–99]	98 [97–99]
HR _(bpm)_ ‡	75.5 [60–87]	81.5 [62.5–90]	90 [75.2–100]^∗∗^	103 [92–103]^∗∗^	97 [85–102]^†^	101 [96-121]^†^	109 [97-132]^∗∗,†^	129 [112-147]^∗∗^,^†^

*Data presented as median and 25–75% interquartile range. QB, quiet breathing; Ti, inspiratory time; Te, expiratory time; Ttot, total time of the respiratory cycle; Exp. Flow, mean expiratory flow; V_*RCp*_/Ti, shortening velocity index of inspiratory ribcage muscles; V_*AB*_/Ti, shortening velocity index of the diaphragm; V_*AB*_/Te, shortening velocity index of the diaphragm; %SpO_*2*_, oxygen saturation; Bpm, beats per minute; L/min, liter per minute; sec, seconds; Intergroup *post hoc* analysis, ^†^*P* < 0.05, ^††^*P* < 0.005 compared to controls; Intragroup *post hoc* analysis, ^∗∗^*P* < 0.005, ^∗∗∗^*P* < 0.001 compared to QB moment; ^‡^Parametric data distribution*.

**FIGURE 2:**
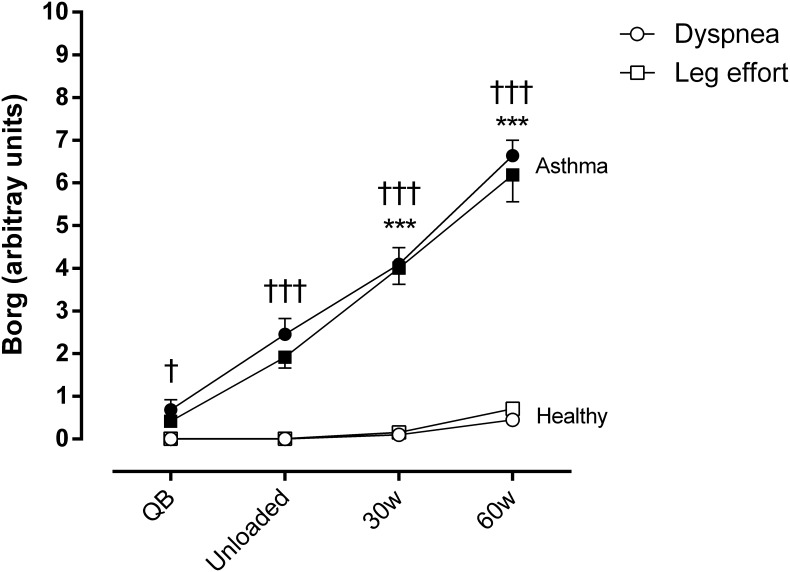
Borg score for dyspnea and sensation of effort in the legs during quiet breathing (QB), unloaded cycling, and 30 and 60 watts of workload in both asthmatic and healthy subjects. Data are shown as mean and SE. Circles, dyspnea; squares, leg effort; ^†^*P* < 0.05 and ^†††^*P* < 0.001 compared to healthy subjects; ^∗∗∗^*P* < 0.001 compared to QB moment.

Regarding dyspnea and sensation of leg effort, significantly higher values were reported by asthmatic subjects both at rest and during exercise (**Figure 2**).

### Chest Wall Compartmental and Operational Volumes

A *post hoc* analysis, considering the calculated within–between effect sizes for V_T,CW_, showed a statistical power (*1-*β) = 1.00 for this study.

Regarding compartmental tidal volumes, the lower tidal volume observed at rest in asthmatic was due to RCa and AB compartmental volumes. During exercise, no significant differences were found, with the exception of RCa that resulted lower at all levels (Cohen’s *f* = 1.37). No significant differences were found in percentage contributions to total tidal volume of the different compartments, both at rest and exercise (**Table 3**).

**Table 3:** Compartmental volumes and percentage of contribution to chest wall volume.

	Healthy				Asthma			
	
	QB	Unload	30 watts	60 watts	QB	Unload	30 watts	60 watts
V_RCp__(L)_	0.264 ± 0.11	0.333 ± 0.10	0.389 ± 0.11^∗∗∗^	0.506 ± 0.13^∗∗∗^	0.214 ± 0.13	0.252 ± 0.09	0.369 ± 0.14^∗∗∗^	0.390 ± 0.17^∗∗∗^
V_RCa__(L)_	0.122 ± 0.03	0.185 ± 0.05^∗∗∗^	0.228 ± 0.06^∗∗∗^	0.281 ± 0.07^∗∗∗^	0.085 ± 0.03^†^	0.019 ± 0.04^††^	0.155 ± 0.04^∗∗∗,†^	0.176 ± 0.07^∗∗∗,††^
V_AB__(L)_	0.359 ± 0.10	0.638 ± 0.25^∗∗∗^	0.731 ± 0.30^∗∗∗^	0.842 ± 0.30^∗∗∗^	0.266 ± 0.10^†^	0.418 ± 0.17	0.557 ± 0.20^∗∗∗^	0.644 ± 0.23^∗∗∗^
%RCp	34.60 ± 12.34	29.46 ± 9.62	29.70 ± 8.10	31.35 ± 6.67	35.32 ± 12.47	31.22 ± 10.30	33.08 ± 8.49	31.50 ± 8.87
%RCa	17.41 ± 6.50	16.89 ± 5.71	17.56 ± 5.35	17.89 ± 77	15.51 ± 3.56	14.34 ± 5.99	15.08 ± 4.76	14.80 ± 4.88
%AB	47.98 ± 10.77	53.65 ± 10.80	52.75 ± 9.07	50.76 ± 7.54	49.17 ± 11.09	54.44 ± 11.94	51.84 ± 10.44	53.70 ± 10.86

*Data presented as mean value and SD. QB, quiet breathing; V_*RCp*_, pulmonary ribcage volume; V_*RCa*_, abdominal ribcage volume; V_*AB*_, abdominal volume; %, percentage of contribution; Intergroup *post hoc* analysis: ^†^*P* < 0.05, ^††^*P* < 0.005, compared to controls; Intragroup *post hoc* analysis: ^∗∗∗^*P* < 0.001 compared to QB moment*.

**FIGURE 3:**
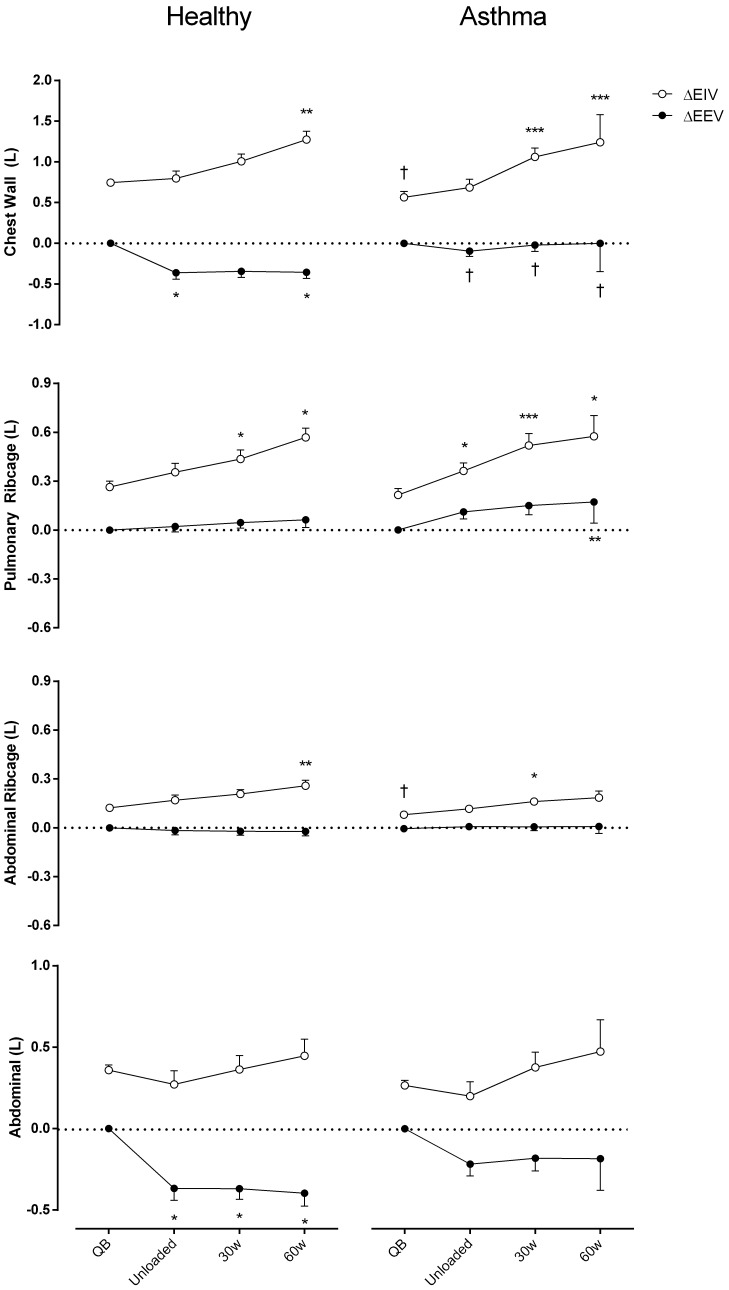
Chest wall and compartmental operational volumes during quiet breathing (QB), unloaded cycling, 30 and 60 watts of workload, and recovery moments (Rec) in both asthmatic and healthy subjects. Data are shown as mean value and SE. Open circles, changes in end-inspiratory volume [ΔEIV]; closed circles, changes in end-expiratory volume [ΔEEV]; L, liters; ^†^*P* < 0.05 compared to healthy; ^∗^*P* < 0.05, ^∗∗^*P* < 0.005, and ^∗∗∗^*P* < 0.001 compared to QB moment.

**Figure 3** shows EEV and EIV variations of the CW and its different compartments compare to QB. The increase of total EIV was due to the two ribcage compartments both in controls and asthmatics. In healthy controls, during exercise the total EEV significantly decreased and this was entirely due to the AB compartment (Cohen’s *f* = 1.23). Conversely, in asthmatic during exercise, the total EEV did not change. This was due to the simultaneous decrease and increase of EEV in the abdomen and increase of EEV of the pulmonary ribcage (Cohen’s *f* = 0.82), with no changes in EEV of RCa.

### Asynchrony

**FIGURE 4:**
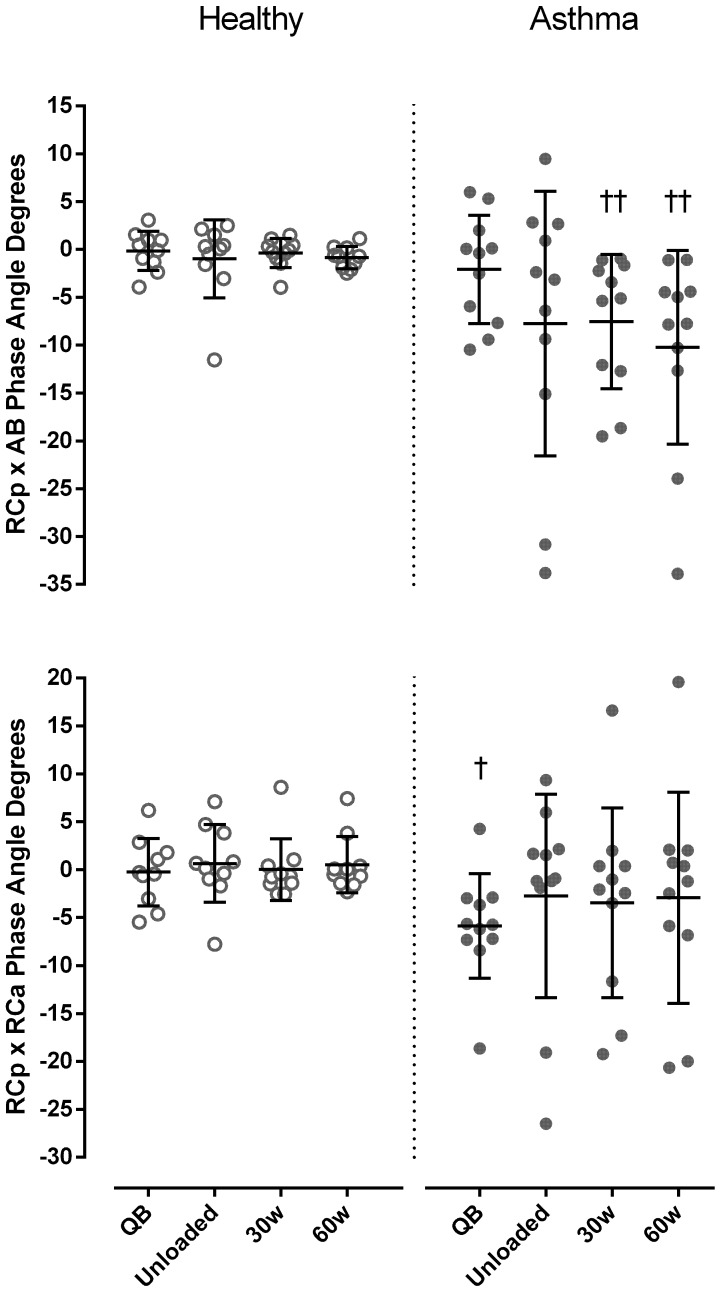
Phase angle degrees between pulmonary ribcage (RCp) and abdominal (AB) (upper panel) and between RCp and abdominal ribcage (RCa) compartments (bottom panel) in both healthy (open symbols) and asthmatic subjects (closed symbols) at rest and during exercise. Data are shown as median and 25–75% interquartile. ^†^*P* < 0.05 and ^††^*P* < 0.005 compared to healthy.

In both healthy subjects and asthmatics, no significant differences were found in θ at different levels. Nevertheless, a significantly more negative RCp-AB phase angle during exercise (*p* < 0.005, Cohen’s *d* = 0.73), and RCp-RCa phase angle at rest (*p* < 0.05, Cohen’s *d* = 0.58) was found in asthmatics compared to controls (**Figure 4**).

**FIGURE 5:**
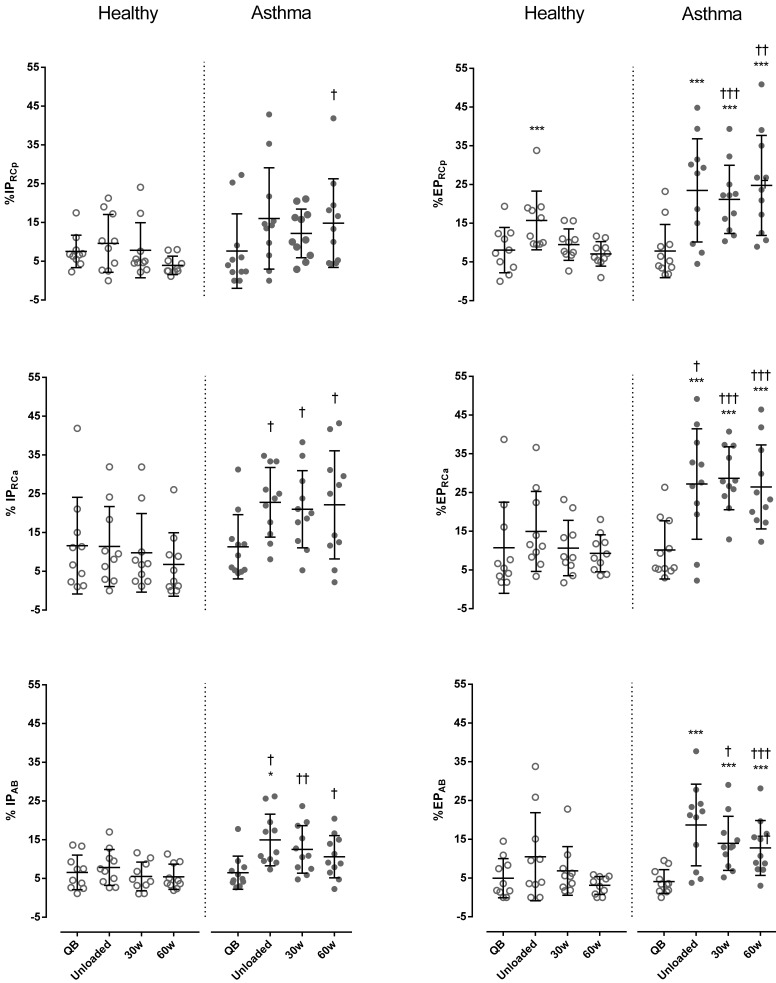
Percentage of inspiratory (%IP) and expiratory times (%EP) of the pulmonary ribcage (RCp) (upper panels), abdominal ribcage (RCa) (middle panels), and abdominal (AB) (lower panels) compartments in both healthy (open symbols) and asthmatic subjects (closed symbols) at rest and during exercise. Data are shown as median and 25–75% interquartile. ^∗^*P* < 0.05 and ^∗∗∗^*P* < 0.001 compared to QB moment; ^†^*P* < 0.05, ^††^*P* < 0.005 and ^†††^*P* < 0.001 compared to healthy subjects.

Regarding paradoxical times, during exercise IP_RCa_ and IP_AB_ were always significantly higher in asthmatic subject than in controls, while IP_RCp_ was significantly higher only at 60 watts (*p* < 0.05, Cohen’s *d* = 0.66). Differently than controls, in asthmatics %EP significantly increased during exercise compared to resting conditions, reaching values significantly higher than controls at 30 and 60 watts in all compartments (**Figure 5**).

**FIGURE 6:**
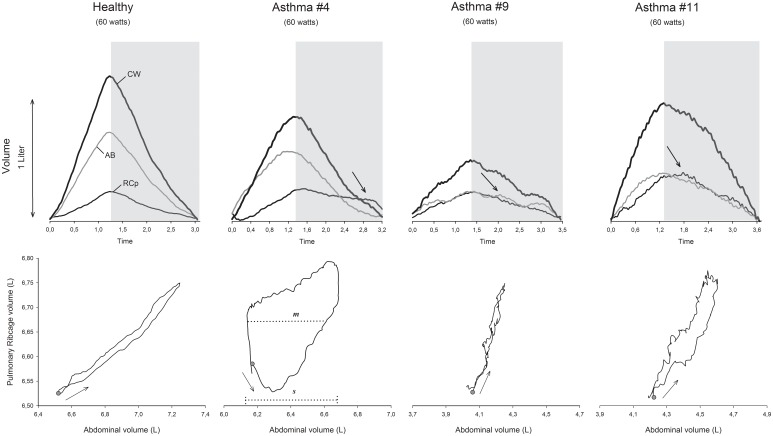
Representative tracings of the normalized chest wall (CW), pulmonary ribcage (RCp), and abdominal (AB) volumes during 60 watts of workload in a cycle-ergometer of 1 healthy and 3 different asthmatic subjects, as well as the respective Lissajous figures. Gray selection, expiratory phase; arrows inside the gray selection, post-inspiratory activity of the pulmonary ribcage (#4 and #11) and diaphragm (#9) muscles; Lissajous loop arrows, direction of the compartmental expansion; gray dots, beginning of inspiration; m, line parallel to the signal of the *X*-axis at 50% of the pulmonary ribcage tidal volume; s, volume of the abdominal tidal volume; L, liters; θ, phase angle; °, degrees.

Representative tracings of within-breath thoracoabdominal volume variations during exercise of one healthy and three asthmatic subjects with the corresponding RCp vs. AB dynamic loops are shown in **Figure 6**.

### Association Between Respiratory Variables

The regression analysis showed a significant association between FEV_1_ and all asynchrony variables (*r* = 0.999; *r*^2^ = 0.987, and *p* = 0.001). The following model was determined:

FEV_1_ (L) = 2.987 + (0.363 × IP_RCp_) + (-0.056 × IP_RCa_) + (-0.283 × IP_AB_) + (-0.282 × EP_RCp_) + (0.065 × EP_RCa_) + (0.277 × EP_AB_) + (-0.042 × RCp-RCa phase angle) + (0.048 × RCp-AB phase angle).

Results for the other regression analysis showed a significant association between dyspnea and asynchrony variables (*r* = 0.739; *r*^2^ = 0.546, and *p* = 0.002). The following equation was found:

Dyspnea = 4.302 + (-0.241 × IP_RCp_) + (0.252 × EP_RCp_) + (0.108 × RCp-RCa phase angle).

## Discussion

The present study provides the first comparison of breathing pattern, CW volume variation, and asynchrony at rest and during mild exercise intensities in asthmatic without exercise-induced bronchoconstriction and healthy subjects. Since different workloads were achieved by all subjects, we decided to analyze and report data until 60 watts of workload, which was the workload present in all asthmatic subjects. In addition, isoventilation analysis was not performed given the fact that during exercise minute ventilation was statistically similar between groups.

The major findings of the present study were that when compared with healthy, asthmatic subjects exhibit the same minute ventilation and a higher rapid shallow breathing index as a result of an increase in respiratory rate during mild exercise. At the same time, DH of the pulmonary ribcage is observed with a significant decrease in inspiratory and expiratory times. In association, a higher thoracoabdominal asynchrony is present during the incremental exercise in asthmatics when compared with controls, namely a significantly more negative phase angle between the pulmonary ribcage and abdominal compartments and significant higher inspiratory and expiratory paradox times. Of these, we can emphasize the IP_RCp_, EP_RCp_, and RCa-RCa phase angle as the thoracoabdominal asynchrony variables that contributed to dyspnea at 60 watts of exercise.

During exercise, healthy humans increase ventilation by increasing both tidal volume and respiratory rate (Aliverti, 2008) and the resistive work is minimized despite high flow rates by intra- and extra-thoracic airway dilatation (O’Donnell et al., 2017). In flow-limited patients, dynamic lung hyperinflation might occur during exercise (Crimi et al., 2002; Vermeulen et al., 2016). Wilkens et al. (2010) described the breathing pattern at rest and during exercise in very severe patients with COPD, pulmonary fibrosis, and cystic fibrosis. These authors showed that patient with both cystic fibrosis and pulmonary fibrosis are characterized by high respiratory rates to cope with the increase in ventilatory demands during exercise, while COPD show a pattern similar to healthy with a similar relative increases of respiratory rate and tidal volume, but with lower duty cycle. In the present paper, the changes in breathing pattern in asthmatic patients are provided as well, showing that during exercise in these patients a substantially increased respiratory rate is adopted to maintain an efficient minute ventilation for gas exchange at low level of exercise, causing a rapid shallow breathing pattern. These findings are in agreement with Crimi et al. (2002), who hypothesized that the increase in EEV without a proportional increase in EIV would constrain tidal volume to remain low at high workloads, thus requiring an increase in respiratory rate to achieve the required increase in minute ventilation.

Minute ventilation and shortening velocity index of respiratory muscles significantly increased during exercise in both groups, but did not differ between groups suggesting a similar central ventilatory drive activity during exercise (Iandelli et al., 2002; Bruni et al., 2012). In addition, as duty cycle and mean expiratory flow were similar both for intra- and intergroup analysis and no changes in spirometric values were observed after the exercise protocol, we can ensure that our asthmatic subjects did not present bronchoconstriction induced by exercise (Parsons et al., 2013). Moreover, the significantly lower expiratory and inspiratory times observed during loaded exercise in asthmatics subjects clearly demonstrate the premature termination of expiration and inspiration, respectively (O’Donnell, 2006). These facts put the respiratory muscles in a disadvantageous portion of their length–tension relationship and may also have contributed, in some extent, to hyperinflation (Filippelli et al., 2003; Aliverti, 2008).

In healthy subjects, the reduction in EEV_CW_ during exercise assists inspiration by optimizing diaphragmatic length and permitting elastic recoil of the CW (Henke et al., 1988). In severe COPD patients, DH can occur during incremental exercise with a progressive increase in EEV_CW_ at the beginning or in the last third period of exercise (Aliverti et al., 2004; Vogiatzis et al., 2005; Georgiadou et al., 2007). In our asthmatic subjects, a significant increase in EEV_CW_ was observed during mild exercise, entirely due to the DH of pulmonary ribcage compartment, suggesting a persistent post-inspiratory action of the inspiratory ribcage muscles (Gorini et al., 1999) and an unfavorable thoracopulmonary configuration obliging the inspiratory muscles to overcome the inward recoil of the respiratory system at the onset of the following inspiration (Gagnon et al., 2014). On the other hand, while RCp accounted for the volume of hyperinflation, the phasic abdominal muscle recruitment during expiration was not fully able to minimize the elastic work of moving the CW (Filippelli et al., 2003), as well as to determine the decrease of EEV_CW_ (Gorini et al., 1999). The altered configuration of the CW compartments at end-inspiration and end-expiration is associated to a high level of thoracoabdominal asynchrony, suggesting an uncoordinated action of the different respiratory muscle groups. Although the mechanisms responsible for these findings are not clear in asthmatic subjects, the following scenario might be considered: during exercise, DH occurs in the pulmonary ribcage modifying its geometry (Gagnon et al., 2014); this reduces the force generation capacity of the inspiratory muscles of the ribcage increasing the abdominal muscle activity to counterbalance the augmented post-inspiratory muscle activity of the RCp during expiration (Filippelli et al., 2003; Vogiatzis et al., 2008; Gagnon et al., 2014), thus initiating inspiration.

The increased IP_AB_ and IP_RCa_ observed in asthmatic during exercise may be attributed to the inability of the abdominal muscles to counterbalance the DH of RCp, while the higher EP_RCp_ and EP_AB_ reinforce the hypothesis of post-inspiratory action of both inspiratory ribcage muscles and diaphragm, respectively. These findings are in agreement with Gorini et al. (1999) who, using optoelectronic plethysmography, observed a sustained post-inspiratory action of the ribcage inspiratory muscles at end-expiration in asthmatic subjects during induced bronchoconstriction. In addition, a similar tonic inspiratory muscle activity during expiration was observed in the study of Kassabian et al. (1982) while using an oscilloscope. However, the asthmatic subjects of this study were acutely ill. Furthermore, as our subjects did not present exercise-induced bronchoconstriction, it appears that DH and post-inspiratory action of the inspiratory muscles do not necessarily need to be accompanied by bronchoconstriction in asthmatics.

Our findings demonstrated that the uncoordinated action of the inspiratory and expiratory muscles was associated with the level of obstruction during exercise in asthmatic patients and that IP_RCp_, EP_RCp_, and RCp-RCa phase angle contributed to a certain extent to dyspnea at 60 watts of exercise. Thus, in general, it can be hypothesized that asynchrony resulted in energy waste and this may have caused an inefficient use of oxygen by the respiratory muscles leading to the increase in dyspnea and leg effort, therefore, resulting in exercise limitation. Interestingly, dyspnea had no association with DH, assessed by the EEV, or any relation with the EIVs of our asthmatic subjects. However, multiple mechanisms likely lead to several distinct sensations of dyspnea in mild asthmatic subjects (Moy et al., 2000) and despite its multifactorial nature (Lougheed et al., 1993), airway narrowing and DH play an important role in the pathogenesis of dyspnea during exercise (Filippelli et al., 2003; Laveneziana et al., 2006). In addition, according to Kosmas et al. (2004), DH is common in stable asthmatics and may contribute to a reduction in exercise capacity in subjects without exercise-induced bronchoconstriction. In our subjects, even with stable asthma, mild exercise provoked a substantial increase in the sensation of dyspnea and leg effort. These responses were more pronounced than those experienced by the study of Moy et al. (2000) and are in line with observations in which DH plays a dominant role on dyspnea (Lougheed et al., 1993; Laveneziana et al., 2006), leading us to believe that DH of the RCp and the thoracoabdominal asynchrony were probably the main factors inducing the increase in perception of effort (Binks et al., 2002; Laveneziana et al., 2006) in our asthmatic subjects. As no discrepant values were observed between both dyspnea and sensation of the effort in the legs, we cannot ensure which of them initiated first to limit exercise capacity (Bruni et al., 2012).

The major limitation of the present study is that data was acquired in two different sites and two different experimental groups were studied, thus any difference in the equipment and/or conditions may have introduced a systematic error. However, the protocol and procedures were strictly followed in both laboratories by previously trained researchers and analysis of data was performed in a blinded way by another author of the study. In addition, data during exercise was presented until 60 watts of workload only, due to the very limited exercise capacity of our asthmatic patients, as well as the inclusion of only healthy males in the control group. Nevertheless, for the first time both the breathing pattern and the operational CW volumes during mild exercise in asthma are described.

## Conclusion

Patients with asthma adopt a characteristic breathing pattern during exercise with high respiratory rate, low tidal volume, DH of the RCp, and post-inspiratory action of the inspiratory ribcage muscles and diaphragm associated to thoracoabdominal asynchrony. Although their relative importance is still to be determined, all these altered features presumably contribute significantly to limit exercise tolerance in asthma.

## Author Contributions

GF, VR, and AA designed and supervised the study. JP and AL collected the data. GF, AS, JP, AL, and AA analyzed and interpreted the data. AS wrote the manuscript. GF, JP, AL, VR, and AA revised the manuscript.

## Conflict of Interest Statement

The authors declare that the research was conducted in the absence of any commercial or financial relationships that could be construed as a potential conflict of interest.
